# Application of Bentonite Clay, Date Pit, and Chitosan Nanoparticles as Promising Adsorbents to Sequester Toxic Lead and Cadmium from Milk

**DOI:** 10.1007/s12011-022-03353-w

**Published:** 2022-07-13

**Authors:** Amany Abdelnaby, Nabila M. Abdelaleem, Elham Elshewy, Ayman H. Mansour, Samar S. Ibrahim

**Affiliations:** 1 Department of Forensic Medicine and Toxicology, Faculty of Veterinary Medicine, Benha University, Toukh, 13736 Egypt; 2 Agricultural Research Center, Animal Health Research Institute, (Benha Branch), Benha, 13512 Egypt; 3 Department of Biotechnology, Agricultural Research Center, Animal Health Research Institute, Dokki,, Giza, 12618 Egypt

**Keywords:** Heavy metals, Food safety, Adsorption, Bentonite clay, Date pit, Chitosan nanoparticles

## Abstract

Evaluating residual lead (Pb) and cadmium (Cd) levels in food products, especially milk, is critical for product safety and quality. In this purview, the current study aims to determine Pb and Cd concentrations in milk using atomic absorption spectrophotometry and compare their values with international standards. In addition, it aims to remove these metals from milk samples using low-cost, naturally occurring materials, such as bentonite, date pit, and chitosan nanoparticles. The ability of potential adsorbents was also investigated using Fourier transform infrared spectroscopy (FTIR), X-ray diffraction (XRD), scanning electron microscopy (SEM), energy-dispersive X-ray (EDX), and transmission electron microscope (TEM). Moreover, their impact on milk’s nutritional properties was considered. The results revealed that most milk samples contained Pb and Cd, with mean values of 0.237 ± 0.179 and 0.041 ± 0.036 mg/kg, respectively. Furthermore, the three possible adsorbents demonstrated high sequestering ability due to their existing functional groups; the adsorption capacity of bentonite to Pb and Cd was 84 and 88%, date pit was 97 and 93%, and chitosan nanoparticles were 82 and 98%, respectively, with no discernible change in milk nutritional contents. In conclusion, the bentonite, date pit, and chitosan nanoparticles were found to be significantly effective and safe in removing hazardous trace elements (Pb and Cd) from contaminated milk.

**Graphical abstract:**

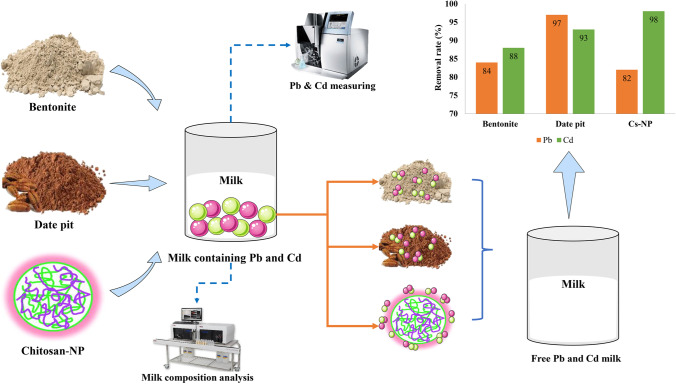

## Highlights

1. An atomic absorption spectrophotometer was used to measure residual lead and cadmium levels in milk.
2. Remediation of metal-contaminated milk using costless and environmentally friendly adsorbents, such as bentonite clay, date pit, and chitosan nanoparticles, was done.
3. There was a difference in the removal effectiveness of bentonite clay, date pit, and chitosan nanoparticles for their removal effectiveness.
4. Fat, protein, and lactose of milk insignificantly changed after adsorbent remediation.

## Introduction

Heavy metals are defined as any metal component with a relatively high density that causes significant health hazards in the body [1]. Due to food safety concerns and potential health risks, among other heavy elements, lead (Pb) and cadmium (Cd) in milk have growing interest since they are broadly spread in the environment, and their accumulation in food poses a significant hazard due to their long-term toxicological implications [2–4].

Pb is responsible for inducing multiple malfunctions and injuries to the central and peripheral nervous system, renal, hematopoietic, and immune systems, as well as memory loss [5, 6]. Children are particularly vulnerable to Pb, as they can absorb a more significant amount than adults, resulting in impaired mental development and behavioral problems. Hyperactivity, lowered immunity, and anemia are also some of the other side effects of Pb [1, 7]. Furthermore, Cd is harmful and non-essential to public health, primarily accumulating in the kidneys and liver. The metal and sewer industries are the most significant sources of Cd exposure [8]. It mainly causes toxicity in the kidneys, liver, brain, lungs, heart, central nervous system, and testicles. In addition, Cd can lead to osteoporosis, anemia, anosmia, non-hypertrophic emphysema, eosinophilia, and persistent rhinitis [5, 7, 9, 10]. Moreover, the International Organization for Cancer Research acknowledges Pb and Cd as human carcinogenes [11, 12].

In order to save human health, it is critical to analyze the residual concentrations of Pb and Cd in milk to monitor these hazardous metals’ levels in products. Various techniques are utilized to extract heavy metal ions from various samples, like chemical precipitation, ion exchange, and reverse osmosis [7, 13]. Adsorption has recently emerged as one of the promising approaches, owing to its simplicity, low cost, and efficiency in heavy metal ions extracted from several kinds of resources [14].

Numerous low-cost adsorbents have already been used among these bentonite clays, corresponding to the smectite clay group [13, 15]. They are also distinguished by their 2:1 structure of two tetrahedral sheets and one octahedral sheet sandwiched in between. It is known to be an effective adsorbent, mainly owing to the net negative charge on the surface generated by isomorphic replacements inside the octahedral sheets, its natural abundance, increased sorption potential, and chemical and mechanical stability [16]. Therefore, Bentonite clay was investigated as an ion exchanger and/or adsorbent due to its structure [13, 17].

Recent research trends attempt to expand the utilization of agricultural waste adsorbents, like date pits, to extract a wide range of environmental contaminants due to their considerable adsorption capability and low cost [18]. Date pit powder is qualified for eliminating trace levels of toxic metal ions with different functional groups, i.e., hydroxyl, phenolic, carboxylic acid, ester, carboxylate, and amino, which can aid as spots for metal ion adsorption [19]. The efficient use of these low-cost sorbents is primarily attributed to functional oxygenated groups found in lignocellulosic substances, including cellulose and lignin units [20].

Chitosan nanoparticles (Cs-NP), colloidal solid particles ranging from 1 to 1000 nm, have piqued the interest of researchers due to their nano size, wide surface area, biodegradability, and remarkable biocompatibility [21]. These characteristics contributed to their high adsorption potential and reactivity, which are both beneficial for extracting heavy metal ions [22]. It is prepared from low-cost, self-degrading, non-toxic, and environmentally friendly biopolymer chitosan. Chitosan (Cs) is a polysaccharide derived from natural chitin present in the exoskeletons of many crustaceans. After cellulose, it is the second most prevalent natural biopolymer [23].

Since milk is a significant contributor to heavy metal contamination, remediation of metal-contaminated milk has received extraordinarily little attention. We used low-cost, naturally derived adsorbents to ensure the product’s safety for human consumption. Noteworthy, bentonite clay, date pit, and Cs-NP have all been employed as effective adsorbents for extracting heavy metal ions from water. However, their potential to adsorb heavy metals in milk-contaminated samples has yet to be evaluated.

Consequently, we aimed to assess Pb and Cd concentrations in milk samples and compare them with the Egyptian and international prescribed limits. The current study was also conducted to investigate the potential metal adsorption capability of bentonite clay, date pit, which was prepared using simple minimal physical pretreatment (heat roasting), and Cs-NP using FTIR, XRD, SEM, EDX, and TEM aside from their effect on fat, protein, and lactose, which are the crucial milk components.

## Materials and Methods

### Chemicals

The substances utilized in this experiment were all analytical reagent grade, nitric acid (65%) and hydrogen peroxide (30%) from Merck, USA. The standards of Pb and Cd were obtained from Sigma (St. Louis, MO, USA). For the preparation of working solutions, the standard solutions were diluted with 0.1 M of HNO_3_. In all dilution procedures, double dH_2_O was used.

### Chelating Adsorbents

Bentonite clay Al_2_O_3_.4 (SiO_2_) H_2_O was purchased from PIOCHEM, Egypt. Date fruits (*Phoenix dactylifera* L.) were purchased from local marketplaces, while Cs-NP was obtained from Nano Gate, Egypt. Foremost, date pit powder was prepared based on the method described by Al-Ghouti et al. [24] by performing the following steps; Date fruits were washed, and pits were easily detached manually. Contaminants were removed from date pits by rinsing those multiple times in dH_2_O. They were dried for 2 h at 65 °C in an oven to remove moisture. Subsequently, dry pits were roasted for 3 h in an oven at 130 °C. The prepared roasted pits were transferred to a coffee machine after being crushed, resulting in coarse to fine particles.

### Samples Collection and Preparation

A total of 35 milk samples were randomly obtained from local farms, independent farmers, and dairy shops in Egypt’s El-Qalyubia governorate (15 raw milk and 20 pasteurized cow’s milk). Pre-washed nitric acid polyethylene containers were used to collect samples. The samples were immediately sent to the laboratory in an ice-packed cooler and were kept at − 20 °C until analysis. For confidentiality, the names of the brands and local markets where samples were obtained were not revealed.

Milk samples were prepared by the acid digestion procedure as described by Ahmad et al. [25]. Briefly, all glassware was first cleaned with a 10% solution of HNO_3_, and after that, it was rewashed with dH_2_O. On a hot plate, 10 mL milk was digested with a 1:3 mixture of H_2_O_2_ and HNO_3_. The samples were then boiled till their volume was decreased to 2 mL. Brown fumes appeared, signaling the completion of organic matter oxidation. The 2 mL sample solution was diluted with dH_2_O (20 mL) to get a clear solution. The same procedure was used for the blank digests in which the samples were replaced with double-dH_2_O. The beaker content was brought with dH_2_O to the appropriate volume and examined using the graphite furnace AAS.

### Assessment of Pb and Cd in Milk Samples

Pb and Cd concentrations in digested samples were determined using the method specified in AOAC [26] by graphite furnace atomic absorption spectrophotometer SensAA (SHIMADZU, Kyoto, Japan) equipped with a hollow cathode lamp at 283.3 and 228.8 nm wavelength, and injection volume of 20 and 10 µL, respectively. Concentrations of Pb and Cd were expressed in mg/kg.

### Characterization of Chelating Adsorbents

#### FTIR Analysis

FTIR spectra of the adsorbents were captured using Nicolet iS50 Thermo Scientific Spectrometer with KBr-pellet in 4000–400 cm^−1^ range for evaluating the surface functional groups of the adsorbents.

#### X-ray Diffraction Analysis

XRD (Bruker D8 Advance, Bruker, USA) was utilized to examine adsorbents’ structural and elemental properties to evaluate their mineralogical composition, influencing their physicochemical properties. The X-ray wavelength was set to 1.54060 A°, and the diffraction peaks were scanned between 2θ = 0° and 80°.

#### SEM Analysis

SEM (JEOL model JSM-6390LA) was applied to identify the pore structures, surface morphologies, topography, shapes, and composition of the substances under analysis. Prior to SEM analysis, samples were coated with gold powder to make them conductive.

#### Energy-Dispersive X-ray Spectroscopy Analysis

Energy-dispersive X-ray analysis (EDX) (Quanttax-EDX, Bruker, Germany) was performed along with SEM to determine the elemental composition of the adsorbents.

#### TEM Analysis

TEM (JEOL, JEM-2100, Electron Microscope) was used to determine the particle form, size, structure, and morphology. The samples were sonicated ultrasonically after being distributed in dH_2_O.

### Chelating Adsorption of Pb and Cd from Milk Samples

The adsorption procedures were carried out at ambient room temperature (25–27 °C). The adsorption assay was accomplished as follows: dividing the fixed volume of previously tested milk samples (20 samples) contaminated with Pb and Cd ions into triplicates (50 mL) and putting them in polyethylene plastic bottles along with each sorbent dose separately.

### Bentonite Clay Adsorption Procedures

A 50 mL of each milk sample was treated with 5 g bentonite clay [15]. The solid–liquid mixture was then violently mixed for 30 min at a speed of 40 cycles per min using the Stuart SSL1 Orbital Shaker. They were then centrifuged for 5 min at 3000 rpm to separate the bentonite clay.

### Date Pit Adsorption Procedures

As illustrated by Samra et al. [27], for 60 min, 50 mL of milk samples was combined with 0.3 g of prepared roasted date pit powder and agitated at 250 rpm. The samples were then filtered to remove any remaining date pits.

### Cs-NP Adsorption Procedures

Mixing 50 mL milk with 0.25 g Cs-Np [28], which was prepared as reported by Hasanin et al. [29], samples were then shaken for 120 min at 180 rpm before filtration. Following that, the concentration of residual Pb and Cd ions in milk samples treated with bentonite clay, date pit, and Cs-Np was determined using graphite furnace AAS. The formula was used to calculate the sequestering efficiencies of bentonite clay, date pit, and Cs-Np in terms of % removal as follows:

1 $$\mathrm{\%\;Removal }=\frac{\mathrm{Ci}-\mathrm{Cf}}{\mathrm{Ci}}\times 100 [19]$$

wherever C_i_- expresses an initial concentration of heavy metals, and C_f_—represents a final concentration of heavy metals.

### Chemical Analysis of Milk Composition

Finally, the nutritional milk components, fats, proteins, and lactose, were measured before and after the addition of adsorbents using mid-infrared FTIR (Milkoscan™ FT1) to investigate the effect of bentonite clay, date pit, and Cs-Np on the chemical properties of milk [15].

### Statistical Analysis

Mean ± standard deviation (SD), minimum, and maximum levels were expressed as concentrations. Statistical analysis was performed using SPSS ver., 22 (Chicago, IL, USA). One-way (ANOVA) and Duncan’s multiple range analysis were utilized to assess the substantial variations in the measured characteristics. The level of statistical significance was determined at *P* < 0.05.

## Results and Discussion

### Assessment of Pb and Cd in Milk Samples

Pb and Cd are non-biodegradable elements that are of great concern due to their adverse effects on human health because they are easily transported through food chains and have no fundamental biological role known to serve [3]. Therefore, internationally, control of these components is a high priority. Milk is contaminated with toxic heavy metals, whether by food and water or the investigated manufacturing and packaging processes [1, 30].

The measured levels of Pb and Cd in collected milk samples are depicted in Table 1. The results revealed that 94.3% of the total milk samples contained Pb with a mean value of 0.237 ± 0.179 mg/kg and a range of 0.008–0.644 mg/kg. According to the findings, in 71.4% of the samples evaluated, the concentration of Cd varied from 0.001 to 0.095 mg/kg, with a mean of 0.041 ± 0.036 mg/kg. Accordingly, the highest occurrence of Pb and Cd was, therefore, higher in pasteurized milk samples than in raw ones.

**Table 1:** Concentrations of Pb and Cd (mg/kg) in the examined milk samples

Element	Milk sample type	No. of samples	Positive samples		Min	Max	Mean ± SD
			No	%			
Pb	Raw milk	15	14	93.3	0.010	0.557	0.270 ± 0.18
	Pasteurized milk	20	19	95	0.008	0.644	0.212 ± 0.18
	Total	35	33	94.3	0.008	0.644	0.237 ± 0.179
Cd	Raw milk	15	7	47.7	0.001	0.067	0.015 ± 0.02
	Pasteurized milk	20	18	90	0.015	0.095	0.06 ± 0.03
	Total	35	25	71.4	0.001	0.095	0.041 ± 0.036

Values are expressed as mean ± SD

*No.* the number of the positive samples and their percentage %, *Min.* minimum, *Max.* maximum, SD standard deviation

In general, high concentrations of Pb in milk can be attributed to environmental factors such as the deposition of pollutants in the atmosphere, waste disposal, vehicle exhaust, and urban effluent [4]. In addition, the provenance of Pb in milk is directly linked to the water as well as the soil and fodder contamination with lead, which may be influenced by polluted water [10]. It should be noted that the Pb levels were very high in milk samples, with 29 samples (82.8%) exceeding the acceptable level of 0.02 mg/kg set by Codex Alimentarius Commission [31] and (EOS) Egyptian standards [32], as depicted in Table 2.

**Table 2:** Number of positive milk samples exceeding the permissible Egyptian and international limits

Element	Egyptian standards^1^		International standards^2^	
	No	%	No	%
Pb	29	82.8%	29	82.8%
Cd	15	42.8%	24	68.5%

^1^The (EOS) Egyptian standards (2007) permissible limits (0.02 mg/kg for Pb and 0.05 mg/kg for Cd)

^2^The Codex Alimentarius Commission (2014) allowable values (0.02 mg/kg for Pb and 0.0026 mg/kg for Cd)

Similar to Pb, the existence of Cd in milk can be caused by natural or anthropogenic causes, such as fertilizers and atmospheric accumulation in soils [4]. Likewise, all Cd-polluted samples suggest the presence of potential fertilizer applications, coal production, and fossil fuel burning as sources of pollution [33]. The Cd accumulation in plants, such as animal feed, has grown nearby industrial locations, mining waste storage areas, and places with high traffic volumes, translocating the Cd ion into the tissues of the animals and, therefore, into the milk [8]. Indeed, the pasteurized milk samples demonstrated elevated occurrence levels than the raw milk samples due to milk contact with heavy metal ions in processing, storage, and transport instruments [30]. The determined levels of Cd observed in 24 samples (68.5%) in the current study surpassed the permissible limit (0.0026 mg/kg) set by Codex Alimentarius Commission [34]. In addition, according to (EOS) Egyptian standards [32], 15 samples (42.8%) exceeded the acceptable limits (0.05 mg/kg).

The findings under investigation coincide with several studies conducted in Egypt. The Pb concentrations in the milk samples in different provinces were 0.135 to 0.614 mg/kg, as in the El-Sayed et al. [35], while Cd levels were between 0.004 and 0.018 mg/kg. Moreover, Malhat et al. found that the average Pb concentration ranged from 1.850 to 4.404 μg/g, while the Cd concentration was between 0.200 to 0.288 μg/g in the El-Qalyubyia governorate [5]. On the contrary, Pb and Cd levels in cow’s milk in the Beni-Suef governorate were 0.054–0.408 and 0.008–0.104 ppm, respectively [4]. Likewise, El-Bassiony et al. illustrated that the average levels in milk obtained from The New Valley governorate were 0.215 and 0.05 ppm with respect to Pb and Cd, orderly [10]. Similarly, the Pb mean values of 0.33 ± 0.049 and Cd mean values of 0.039 ± 0.007 ppm were observed in raw milk in Alexandria city, according to Abo El-Makarem et al. [36].

### Characterization of Chelating Adsorbents

#### FTIR Analysis

**Fig. 1:**
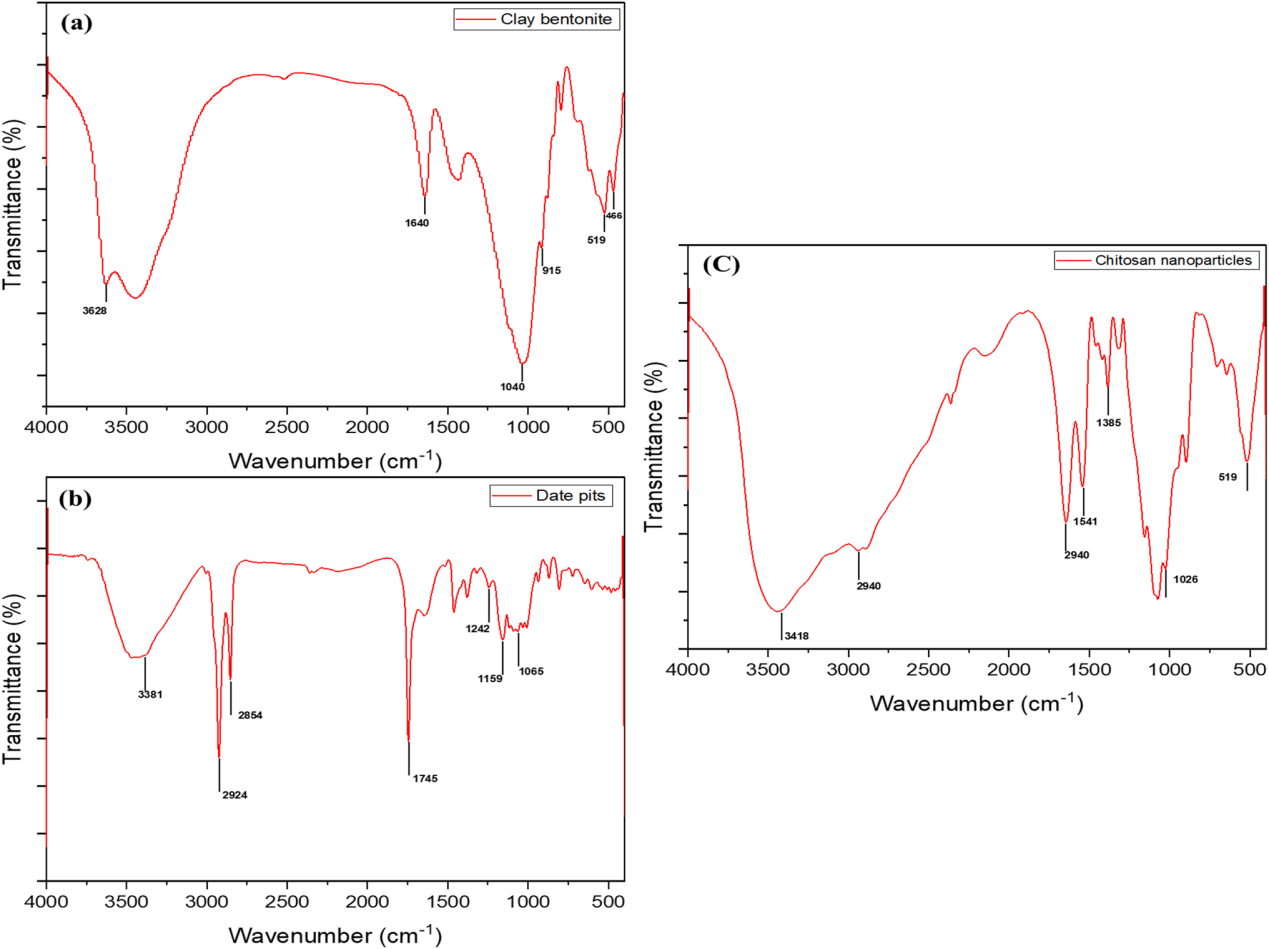
FTIR spectra of bentonite clay (**a**), date pit (**b**), and chitosan nanoparticles (**c**)

The FTIR technique was employed to better understand the adsorption mechanism. FTIR is a valuable tool for characterizing the functional groups on the surface of adsorbents. The FTIR examination of bentonite clay revealed an intense band at 1040 cm^−1^, which is attributed to the silicate (Si–O-Si) structure, as well as additional other bands at 915, 519, and 466 cm^−1^ may be assigned to octahedral (Al- O- Si), (Al-Al-O), and (Si- O- Si) bending vibrations, respectively (Fig. 1). The presence of iron oxide is demonstrated by the combined band at 466 cm^−1^ [16]. It was found that bond O–H deformation of the contained water molecules is related to the small band at 1640 cm^−1^. The band at 3628 cm^−1^ can also be related to OH-stretching bands [37].

As represented in the date pit FTIR spectrum (Fig. 1), a large band of the O–H stretching vibration emerges at 3381 cm^−1^, which may be linked to hydroxyl groups both free and bound (e.g., carboxylic acids, phenols, and alcohols) found in cellulose, lignin, and pectin [38]. Two near bands at 2924 and 2854 cm^−1^ can be related to asymmetric C–H stretching of aldehyde molecules as well as the acetyl and ester groups’ existence in the date pit hemicellulose structure [24]. Carbonyl groups (–COOH and –COOCH_3_), carboxylic acids, or their esters are assigned to the stretching vibration of C = O found at 1745 cm^−1^. The syringe ring and C-O stretch may be related to the absorption band at 1242 cm^−1^ in lignin and xylan. C–O–C vibration and C-O stretch of carboxylic acid, alcoholic, and ester groups of cellulose and hemicellulose may be linked to bands near 1159 and 1065 cm^−1^, respectively [18, 38]. Consequently, date pit FTIR analysis revealed its hemicellulose structure, which is involved in the Pb and Cd adsorption mechanisms.

The FTIR spectrum of Cs-NP revealed major absorption bands at 3418, 2940, 1645, 1541, 1385, 1026, and 519 cm^−1^, respectively (Fig. 1). The identified intense broad absorption band at 3462 cm^−1^ may be due to the stretching vibrations of –NH_2_ and O–H and attributed to molecules’ extra molecular hydrogen bonds [39]. The amount of water found in the Cs-NP indicated that they were hydrophilic, as evidenced by the presence of the – O– H bonding detected between 3200 and 3550 cm^−1^ [40]. The asymmetric stretching vibration of methylene (CH_2_) could explain the absorption band at 2940 cm^−1^ [23]. The bending vibration of N–H generates an absorption band at 1645 cm^−1^ due to Cs and TPP interaction. The bands at 1541 and 1385 cm^−1^ are attributable to an aliphatic nitro compound’s N–O stretching. The primary alcohol C–O stretching vibration had a peak of 1026 cm^−1^. The absorption band at 519 cm^−1^ was found to be connected to bromoalkanes [40]. In general, the FTIR spectrums of three used adsorbents demonstrate the complexity of their structure and their ability to behave as prospective adsorbents, as evidenced by the presence of the functional groups on their surfaces.

#### XRD Analysis

**Fig. 2:**
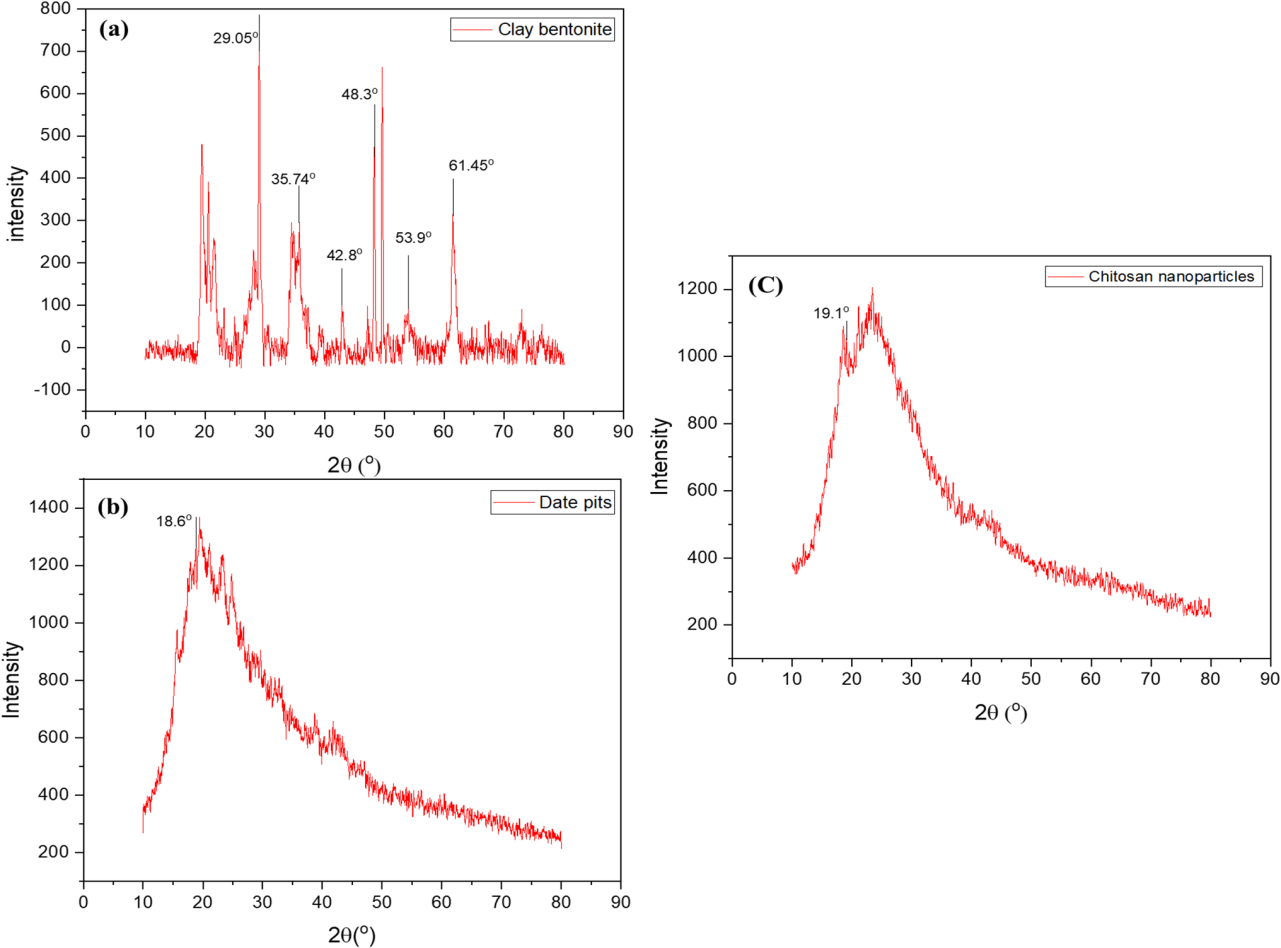
XRD spectra of bentonite clay (**a**), date pit (**b**), and chitosan nanoparticles (**c**)

XRD analysis contributes to identifying the sample physicochemical properties through its mineralogical components and determines the compatibility of each component material [16]. The bentonite clay XRD demonstrated the highest intensity of diffraction peaks (100%) at 2θ values of 29.05° and other diffraction peaks at 2θ values of 61.45 and 35.74° with relative intensities of 43.4 and 45%, respectively, which may be attributed to silicon oxide compound (Fig. 2). Other lower intensive diffraction peaks were observed at 2θ values of 42.8°, which may be attributed to aluminum oxide, and 53.9° and 48.3°, which may relate to magnesium oxide (MgO). These findings revealed that bentonite clay was an aluminosilicate mineral [41].

As demonstrated in Fig. 2, the XRD of the date pit showed no straight horizontal fundamental line and only a few diffraction peaks. With a relative intensity of 100%, the XRD pattern revealed intense diffraction peaks at 2θ values of 18.6°. This study revealed that date pits are mostly amorphous and contain little crystalline materials [38]. The amorphous form of the date pit allows for simple Pb and Cd ions migration to its surface, resulting in excellent metal ions removal [42].

The XRD study of Cs-NP revealed the presence of clearly identified diffraction peaks (Fig. 2). The XRD pattern revealed intense diffraction peaks at 2θ values of 19.1°, with a relative intensity of 100%. Cs-NPs’ XRD pattern is suggestive of an amorphous polymer, which aligns with the findings of previous studies [28, 39, 43] because the Cs-NP structure is made up of dense counter-ions interpenetrating TPP, whereas polymer chains are cross-linked by TPP counter ions. As a result, the Cs-NP’s crystalline structure was disrupted after TPP cross-linking. The decrease in polymer crystallinity improves metal ion sorption and capacity, as reported by Ali et al. [28]. Considering the XRD diagram, we can contend that bentonite clay, date pit, and Cs-NP could adsorb Pb and Cd on their surfaces.

### SEM Analysis

**Fig. 3:**
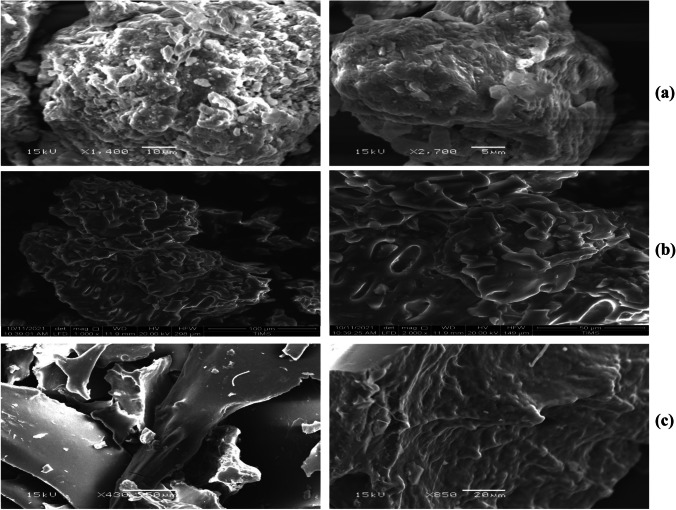
SEM micrograph descriptions of bentonite clay (**a**), date pit (**b**), and chitosan nanoparticles (**c**) at two magnifications

The morphology of the three adsorbents studied in SEM is illustrated in Fig. 3, where the sheet-like structure of bentonite clay is demonstrated in Fig. 3a, and the surface showed a high porosity that increases the contact area and promotes adsorption at active sites of the positively charged ions [13, 16]. It is evident that bentonite clay particles were made up of heterogeneous aggregates of assorted sizes and shapes. These grains form a stack of sheets, indicating clay layers. A little bright crystallite settles on the sample’s surface, which could be made of free silica [37]. In contrast, the SEM micrograph, Fig. 3b, showed a porous surface of the date pit with large pores size and defined structure, thus facilitating the diffusion of particles within the date pit and enhancing the adsorption ability [24]. Generally, most of the constituents have irregular ends with a rough structure, as well as the presence of numerous cavities and holes that allow Pb and Cd ions to be adsorbed. Our findings are consistent with earlier reports [24, 27, 42, 44]. Conversely, the SEM image in Fig. 3c revealed that Cs-NP is intertwined and well dispersed. This form of morphology increases the material’s surface area, making it very convenient for adsorption, by comparing the SEM micrograph descriptions of Cs-NP with previous authors who claimed that Cs-NP seems to have a spherical form [28, 39]. The spherical morphology of Cs-NP was not clearly visible in our SEM image; however, they seemed to have a rod-shaped structure. The current study findings are consistent with Vijayalakshmi et al. [43].

#### Energy-Dispersive X-ray Spectroscopy Analysis

**Fig. 4:**
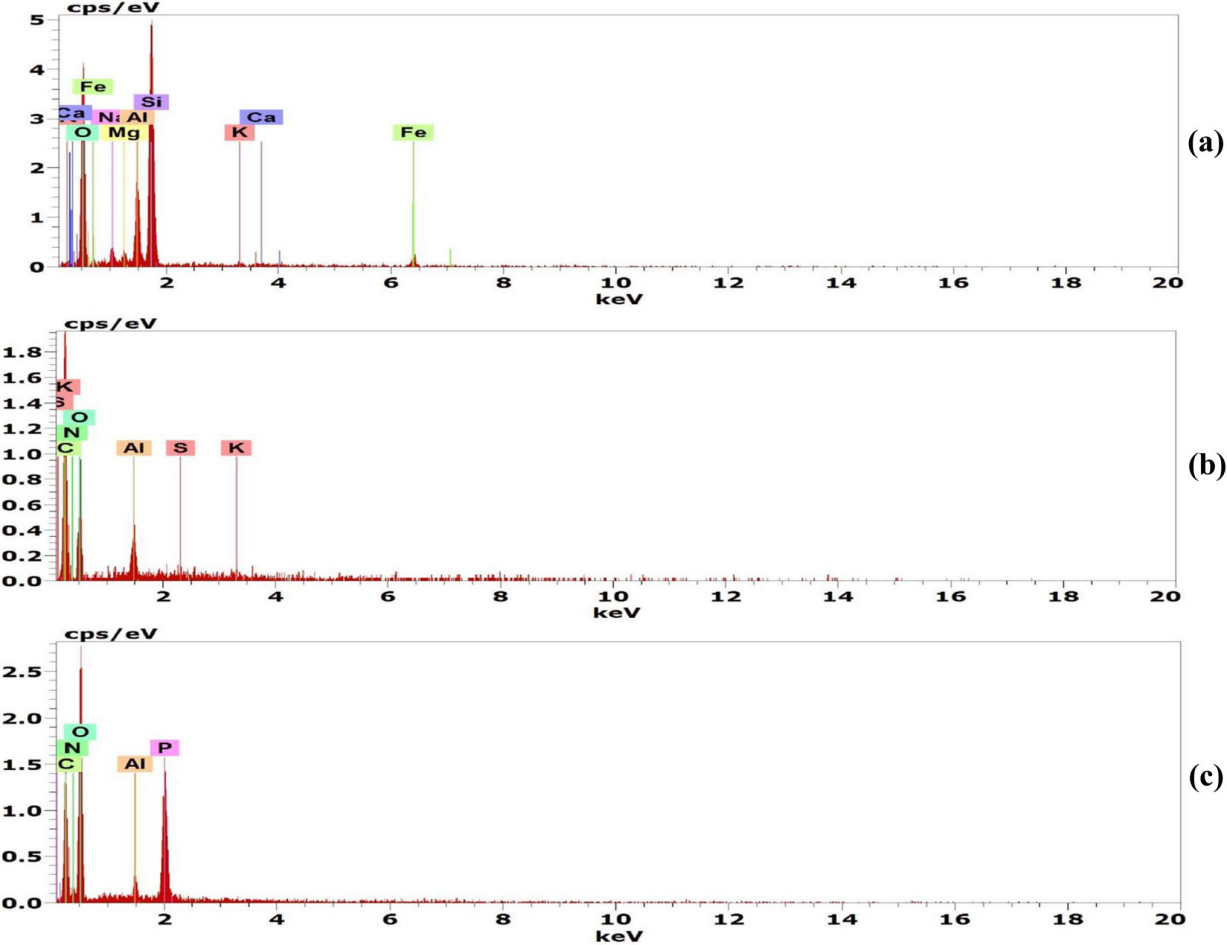
EDX analysis of bentonite clay (**a**), date pit (**b**), and chitosan nanoparticles (**c**)

The elemental compositions of bentonite clay, date pit, and Cs-NP have been studied by EDX. The elemental composition of bentonite clay is shown in Fig. 4 and Table 3, which reveal that the dominant elements are O, Si, and Al in a percentage of 62.9, 28.05, and 10.22%, respectively, confirming the aluminosilicate structure [13]. Na, Mg, K, Ca, and Fe are other exchangeable cations that exist in lower amounts. By ion exchange, these positively charged ions will enhance the Pb and Cd adsorption on the bentonite surface [16]. The spectrum in Fig. 4 demonstrates that the date pit primarily comprises carbon and oxygen, which account for 56.13 and 40.42%, respectively. In addition to the presence of elements, such as aluminum 2.83%, sulfur, and potassium, also exist but at a percentage < 1%. Our results are similar to that of Al-Saad et al. [45]. Consequently, the surface roughness of the date pit observed in SEM may be due to the existence of its main functional groups of different carbon [42].

**Table 3:** EDX elemental analysis of bentonite clay, date pit, and chitosan nanoparticles (Cs-NP)

Adsorbents	Element	Weight %
Bentonite clay	O	62.9
	Na	3.85
	Mg	2.31
	Al	10.22
	Si	28.05
	K	0.47
	Ca	3.20
	Fe	3.06
Date pit	C	56.13
	N	0
	O	40.42
	Al	2.83
	S	0.17
	K	0.45
Cs-NP	C	31.53
	N	8.07
	O	53.55
	Al	0.74
	P	6.11

The elemental compositions of Cs-NP are shown in Fig. 4 and Table 3. The weight percentages of critical elements such as C, O, and N are 31.53, 53.55, and 8.07%, respectively, with the presence of P (6.11%) and Al (< 1%) [23]. These findings demonstrate the presence of amine functional group-containing N and organic matters, like C, in Cs-NP that participate in Pb and Cd adsorption.

#### TEM Analysis

**Fig. 5:**
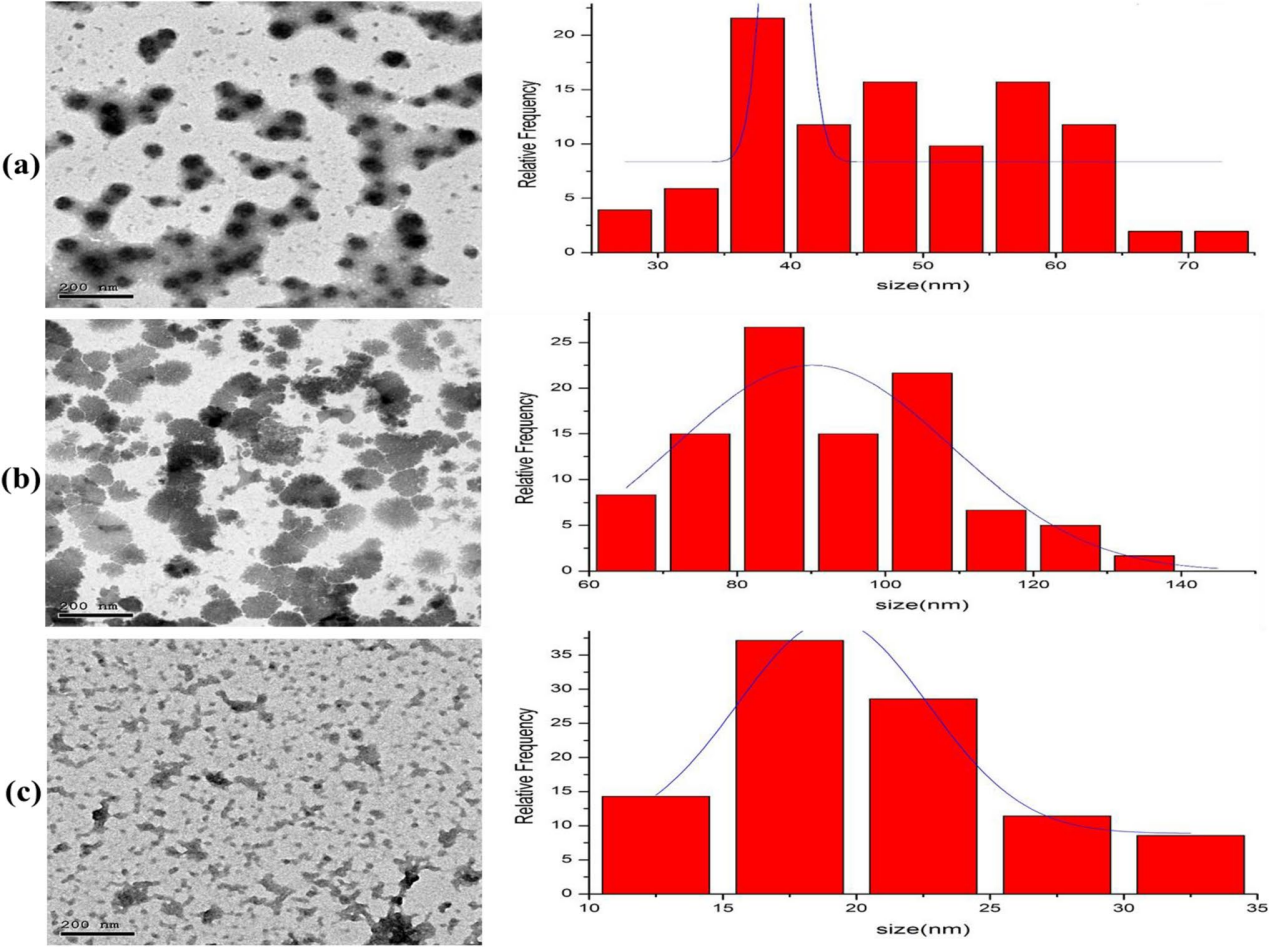
TEM micrograph descriptions of bentonite clay (**a**), date pit (**b**), and chitosan nanoparticles (**c**) with their size histograms

The TEM analysis was used to determine the size, shape, and morphology of the employed adsorbents. Figure 5 demonstrates the typical TEM images of bentonite, date pit, and Cs-NP, with size histograms beside the corresponding TEM images. The TEM images of the bentonite in Fig. 5a revealed a number of clusters of various sizes and forms. The unique interlaying nature and crystalline substrate can be seen clearly. Surface morphology is analogous to that noticed in SEM photos. According to the TEM image findings, the particle size of bentonite was approximately 47 ± 10.4 nm, which are average montmorillonite dimensions[46]. The TEM micrograph Fig. 5b of the date pit revealed substantial porosity and roughness, which was consistent with the SEM results. Due to the presence of cellulose, the date pit’s morphology revealed irregular clusters with distinct particle size. According to histogram measurements, the predominant particle size of the date pit was around 91.9 ± 17 nm [47]. The nearly irregular spherical shape of the CS-NP is clearly visible in TEM micrograph (Fig. 5c). The typical diameter of CS-NP is estimated to be 19.8 ± 5.64 nm. The results obtained are compatible with prior reports [28, 29, 39]. TEM scans revealed that bentonite, date pit, and CS-NP all had an average size under 100 nm. Based on the previously mentioned results, TEM studies suggest that the number of active sites in bentonite, date pit, and CS-NP improves their adsorption abilities.

### Chelating Adsorption of Pb and Cd from Milk Samples

Due to the widespread recognition of the significant and well-known chemical risks of Pb and Cd in milk, the use of decontamination methods that do not change the nutritional content of milk intended for human consumption is essential, primarily focusing on adsorption practices attributed to their simplicity, affordability, accessibility, and efficiency [16].

Table 4 summarizes milk samples’ adsorbent sequestering efficacy for Pb and Cd. The experimental studies showed that bentonite clay could sequester Pb and Cd (84% and 88%) with mean values of 0.059 ± 0.04 and 0.008 ± 0.007 mg/kg, respectively. Based on the screening results, bentonite clay which is aluminum phyllosilicate primary composed of smectite mineral of montmorillonite [17] has been demonstrated to serve as an effective detoxifying agent of Pb and Cd from contaminated milk at approximately 84 and 88%, respectively. This finding can be attributed to the higher adsorption potential of the negative charged clay for the adsorption of positive ions such as Pb^+2^ and Cd^+2^ [13]. The bentonite clay’s aluminosilicate structure, besides its exchangeable cations, has been proven by FTIR, XRD, SEM, EDX, and TEM analysis. It is noteworthy that its removal mechanism could be linked to the chemisorption method, its higher electronegativity, and its composition with a layer of one octahedral sheet and two sheets of silica tetrahedral, which in turn holds a net negative charge as evidence for the broken bonds at the edges of the aluminum–silica units ultimately leading to unsatisfied charges, can be balanced by the exchange of cations [17, 48]. Figure 7 depicts a schematic representation of the proposed adsorption mechanisms between Pb, Cd, and bentonite clay. Our results align with earlier studies in aqueous solution [7, 13, 16], indicating the adsorption ability of bentonite clay to Pb and Cd ions.

**Table 4:** The adsorption of Pb and Cd from contaminated milk samples by bentonite clay, date pit, and Chitosan nanoparticles (Cs-NP) (*N* 20)

Element	No adsorbents	After adsorbents					
	Control	Bentonite clay		Date pit		Cs-NP	
	Mean ± SD	Mean ± SD	Removal %	Mean ± SD	Removal %	Mean ± SD	Removal %
Pb (mg/kg)	0.363 ± 0.12	0.059 ± 0.04	84%	0.012 ± 0.01	97%	0.065 ± 0.06	82%
Cd (mg/kg)	0.065 ± 0.03	0.008 ± 0.007	88%	0.005 ± 0.003	93%	0.001 ± 0.001	98%

Data are expressed as (mean ± SD)

The percentage of Pb and Cd removal was determined compared to controls with no adsorbing agent *SD* standard deviation

**Fig. 6:**
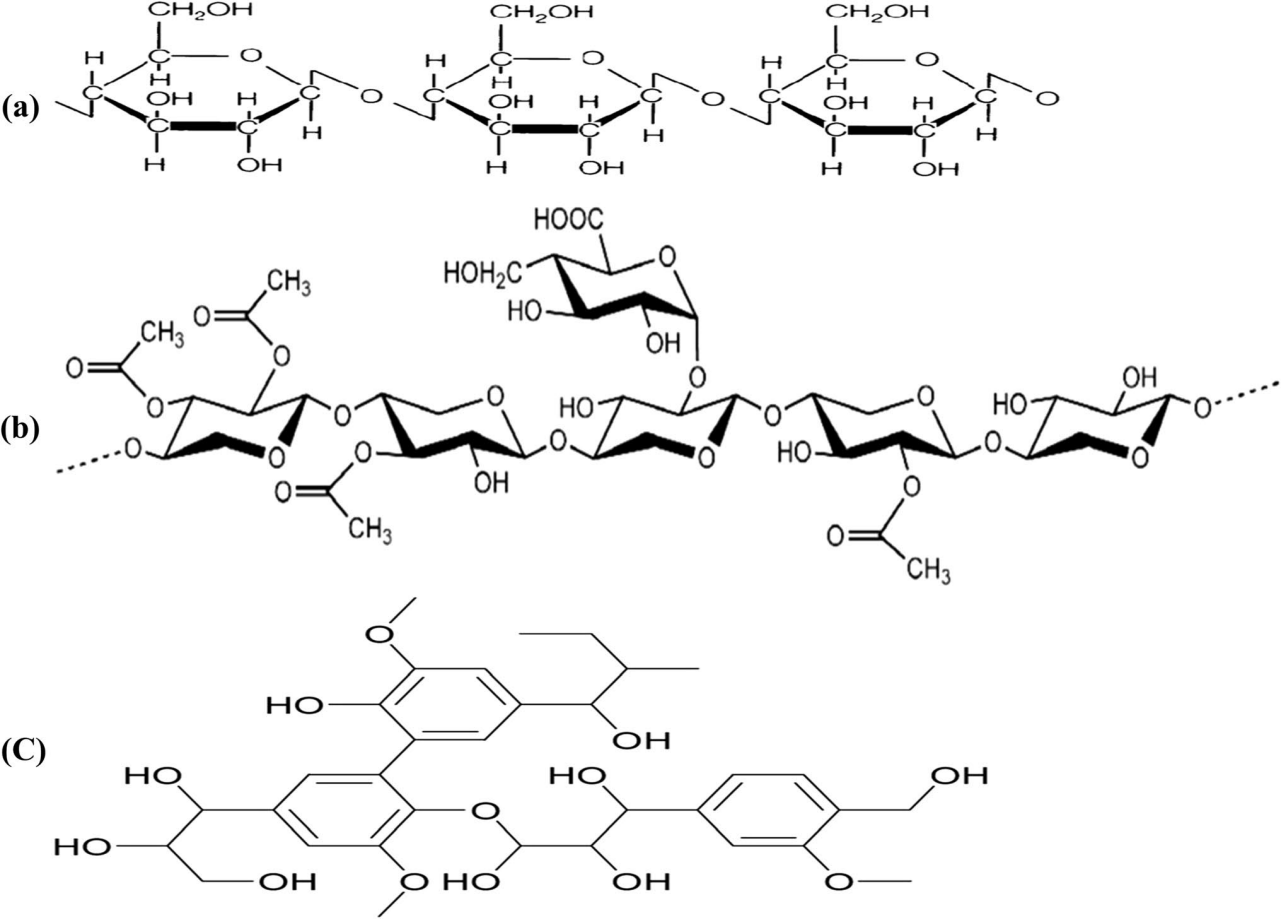
The chemical structure of (**a**) cellulose, (**b**) hemicellulose, and (**c**) lignin (the major components of date pit)

Date pit (Table 4) showed a trend toward removal of Pb and Cd (97 and 93%) with average (0.012 ± 0.1 and 0.005 ± 0.003 mg/kg), respectively. Promisingly, the adsorption studies showed a high potential of date pit for Pb and Cd reduction from milk, where 97 and 93% removal were achieved. The date pits are deemed a waste of zero economic value with possible disposable problems and constitute around 15% of the date fruit weight [24]. It is worth mentioning that the date pit consists primarily of three main components: cellulose, hemicellulose, and lignin [49]. The chemical structure of these components is shown in Fig. 6. Many oxygen functional groups found in lignocellulosic content, including cellulose and phenolic compounds, contribute to the high removal efficiency of date pits, as confirmed in FTIR spectrum (Fig. 1) [44]. Two probable mechanisms of date pit adsorption were described by Al-Ghouti et al. [20], where the first was attaching cellulose and hemicellulose units to lignin units using 2 OH-groups in which lignin binds between and inside both cellulose and hemicellulose units serving as a cementing matrix. In addition, it is suggested that the other adsorption mechanisms can be related to the forces of dispersion, complexation, hydrogen bonding, or electrostatic interactions.

**Fig. 7:**
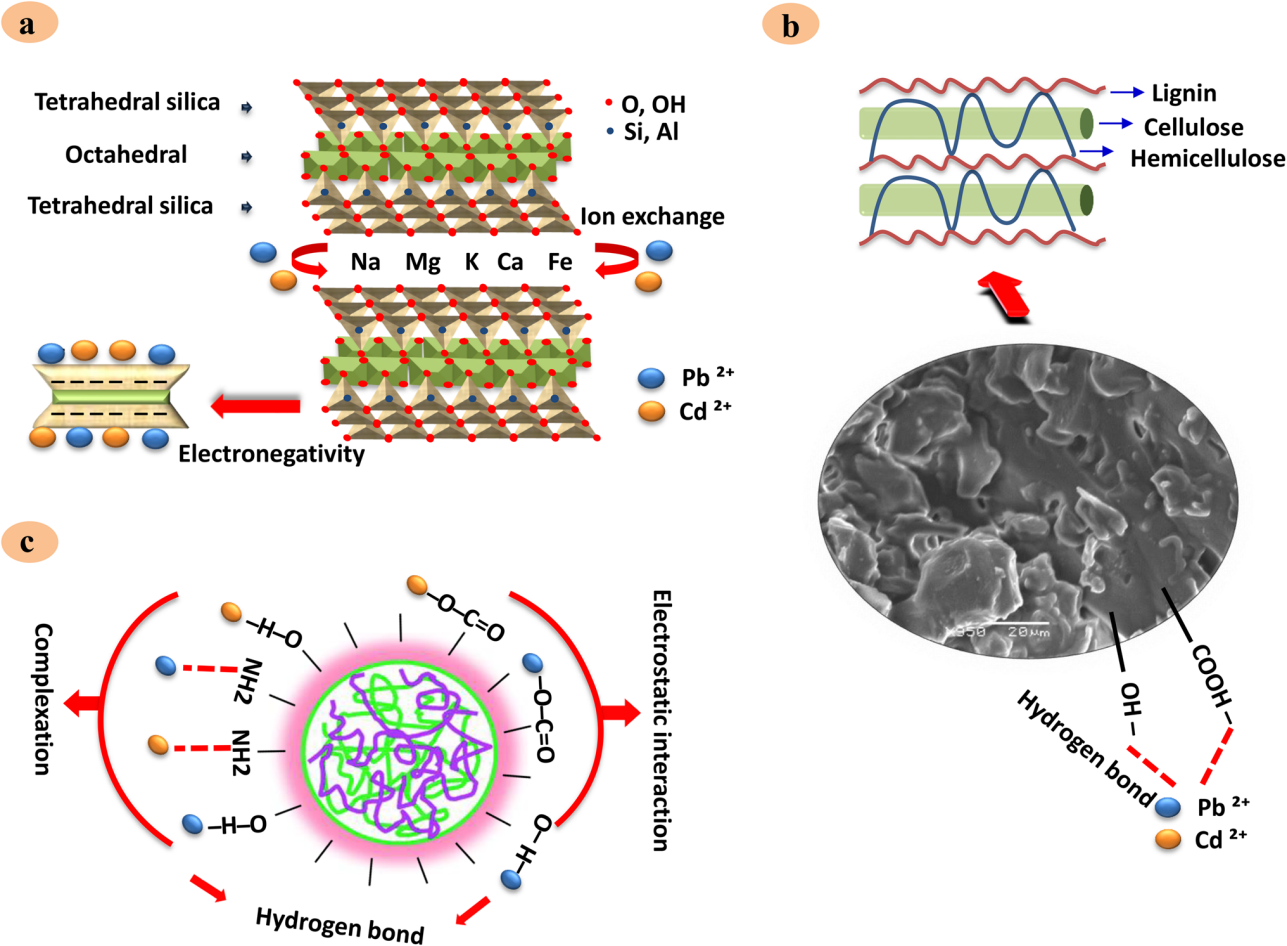
Proposed adsorption mechanisms between (Pb and Cd) and bentonite clay (**a**), date pit (**b**), and chitosan nanoparticles (**c**)

Similarly, Hassan et al. [47] proved that the reactions on the roasted date pit surface correlated with the nuclei of the aromatic rings, which were linked to the roasted date pit’s functional groups, like – OH and – COOH. The proposed mechanism of date pit adsorption is demonstrated in Fig. 7. Notably, there is a paucity of data as well as studies on the utilization of date pits as heavy metal adsorbents from milk. Most research has focused on its potential application as an adsorbent for heavy metals in water. These findings in Table 4 are in harmony with those achieved by Mohamed et al. and Samra et al. [19, 27].

Cs-NP also showed a substantial reduction of Pb and Cd (82 and 98%) with an average of 0.065 ± 0.06 and 0.001 ± 0.001 mg/kg compared to the Pb- and Cd-untreated control samples which have a mean value of 0.363 ± 0.12 and 0.065 ± 0.03 mg/kg, respectively. Furthermore, as depicted in Table 4, Cs-NP, obtained from the amino-functionalized polysaccharide chitosan extracted from the waste shells of crustaceans to improve the surface area for adsorption [50], displayed a remarkable capture of Pb and Cd (82% and 98%). The high adsorption potential of Cs-NP can be elucidated by the sturdy electrostatic binding of its functional groups with metal ions (various carboxylic and hydroxyl), as shown in FTIR (Fig. 1). In addition, this electrostatic interaction between the lone pair of amine functionality (NH_2_) nitrogen atom electrons of chitosan with bivalent (II) Pb or Cd [23]. Alyasi et al. clarified the several adsorptions’ mechanisms with two key techniques of complexation bonding: a complex with one functional amine group and the second type of complexing with two groups of amines and hydroxyls [50]. In this regard, a representative diagram of the adsorption mechanism is shown in Fig. 7. Our findings are congruent with those previous reports [23, 28], which revealed that Cs-NP is a powerful, safe, and reliable substance used as efficient heavy metals adsorbents from an aqueous solution.

### Chemical Analysis of Milk Composition

**Fig. 8:**
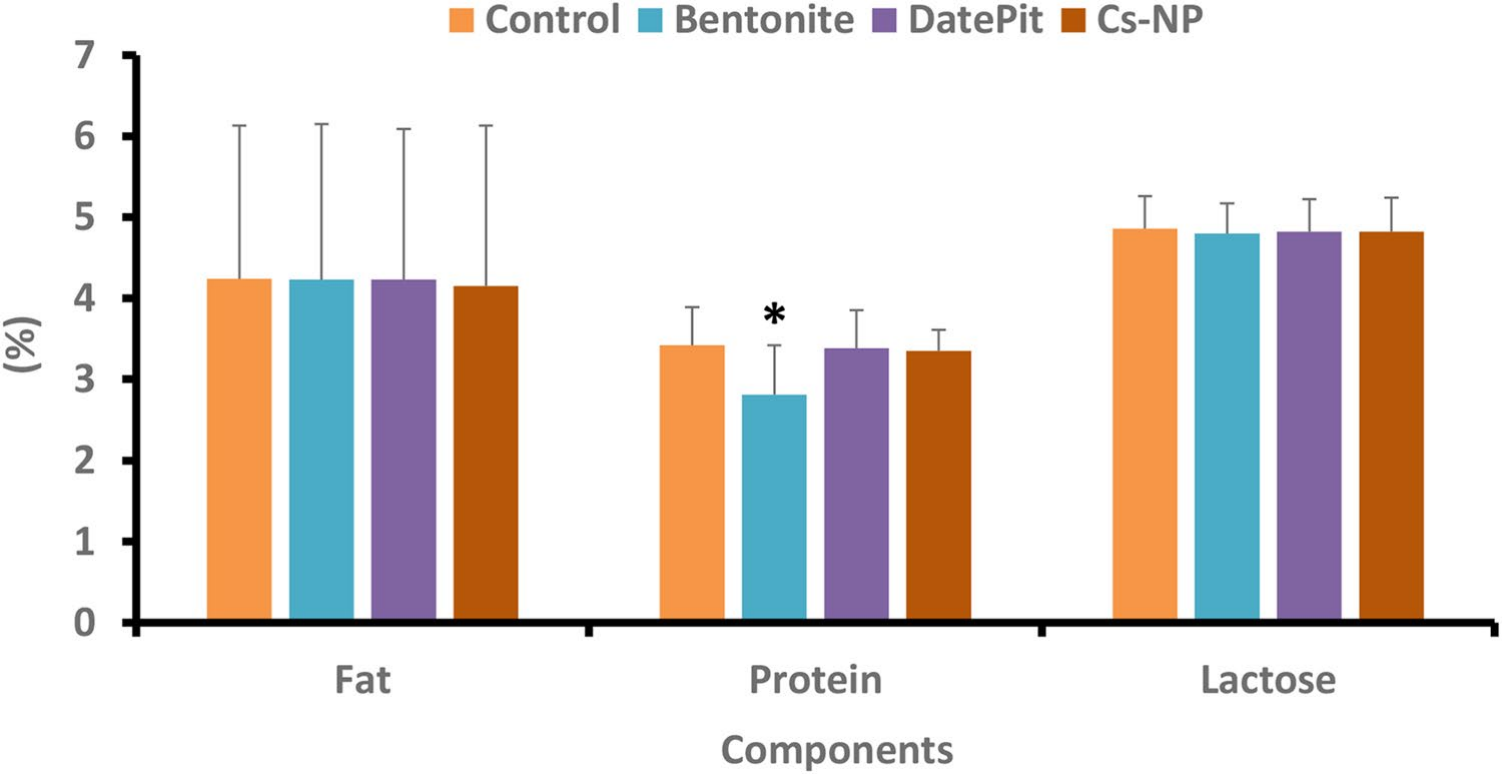
Chemical analysis of the milk composition. The data are represented as (mean ± SD). There were no substantial changes in the chemical parameters of milk, including fat, protein, and lactose, after the milk remediation with bentonite clay, date pit, and chitosan nanoparticles (Cs-NP), except for the slight decrease in protein in the case of bentonite clay

The significant aspect of this research is that the date pit and Cs-NP showed no affinity for milk fat, protein, or lactose, and the nutritional properties of the milk remained unaffected, as demonstrated in Fig. 8. Nevertheless, adsorption treatment with bentonite clay slightly reduces protein content in milk dispersion from 3.42 to 2.81% on a regular basis while maintaining fat and lactose levels. This decrease might be due to milk protein sequestration by bentonite clay, which is encouraged at low pH, as proteins are easily protonated and adsorbed, or it could be connected to the electronegative structure and the functional groups, as demonstrated by FTIR, XRD, SEM, EDX, and TEM studies. Unlike milk proteins, milk fat adsorption is not of concern. Furthermore, milk’s lactose content did not significantly fluctuate after adding bentonite clay [15].

The current study’s findings indicate that bentonite clay, date pit, and chitosan nanoparticles are promising adsorbents for heavy metal removal in milk. Their efficacy in removing heavy metals has been established earlier, particularly in water, with little emphasis on milk [7, 16, 23, 24, 45, 50]. The most significant part of our findings is that we are the first to offer new potential in milk purification by utilizing an efficient, simple, and low-cost heavy metal treatment alternative without any chemical alteration to the tested sorbents or modifying the nutritional profile of the product or the availability of its nutritional component. Consequently, the proposed work adds to our understanding of food safety issues by providing a good reference for heavy metal removal from milk products.

## Conclusion

This research was initially conducted to analyze Pb and Cd content in raw and pasteurized cow’s milk through flame atomic adsorption analysis. Based on the previous findings, we concluded that the Pb and Cd concentrations in most milk samples collected exceed the allowable limits of EOS and Codex Alimentarius Commission. These values mean that the inhabitants of the province of EL-Qalyubia are vulnerable to a potential health risk associated with milk intake. Furthermore, this research aimed to establish a model for evaluating several adsorbents’ efficacy in removing toxic Pb and Cd from milk. The research suggests new effective adsorbents (bentonite clay, date pit, and chitosan nanoparticles) of Pb and Cd from milk dependent on their structures and functional groups, as demonstrated by FTIR, XRD, SEM, EDX, and TEM analysis, as well as the nutritional composition of milk in terms of fat, protein, and lactose, have been preserved using such adsorbents.

## Data Availability

The generated and analyzed datasets during the study are available per request from the corresponding author.

## Abbreviations

AAS: Atomic absorption spectrophotometer
Cd: Cadmium
Cs-NP: Chitosan nanoparticles
Cs: Chitosan
EDX: Energy-dispersive X-ray
EOS: Egyptian Organization for Standardization and Quality
FTIR: Fourier transform infrared
Pb: Lead
SEM: Scanning electron microscope
TEM: Transmission electron microscope
XRD: X-ray diffraction
